# Distinct Molecular Effects of Angiotensin II and Angiotensin III in Rat Astrocytes

**DOI:** 10.1155/2013/782861

**Published:** 2013-02-14

**Authors:** Michelle A. Clark, Chinh Nguyen, Hieu Tran

**Affiliations:** Department of Pharmaceutical Sciences, College of Pharmacy, Nova Southeastern University, 3200 South University Drive, Fort Lauderdale, FL 33328, USA

## Abstract

It is postulated that central effects of angiotensin (Ang) II may be indirect due to rapid conversion to Ang III by aminopeptidase A (APA). Previously, we showed that Ang II and Ang III induced mitogen-activated protein (MAP) kinases ERK1/2 and stress-activated protein kinase/Jun-terminal kinases (SAPK/JNK) phosphorylation in cultured rat astrocytes. Most importantly, both peptides were equipotent in causing phosphorylation of these MAP kinases. In these studies, we used brainstem and cerebellum astrocytes to determine whether Ang II's phosphorylation of these MAP kinases is due to the conversion of the peptide to Ang III. We pretreated astrocytes with 10 **μ**M amastatin A or 100 **μ**M glutamate phosphonate, selective APA inhibitors, prior to stimulating with either Ang II or Ang III. Both peptides were equipotent in stimulating ERK1/2 and SAPK/JNK phosphorylation. The APA inhibitors failed to prevent Ang II- and Ang III-mediated phosphorylation of the MAP kinases. Further, pretreatment of astrocytes with the APA inhibitors did not affect Ang II- or Ang III-induced astrocyte growth. These findings suggest that both peptides directly induce phosphorylation of these MAP kinases as well as induce astrocyte growth. These studies establish both peptides as biologically active with similar intracellular and physiological effects.

## 1. Introduction

Mitogen-activated protein (MAP) kinases constitute a superfamily of serine/threonine protein kinases involved in the regulation of a number of intracellular pathways associated with cellular growth, apoptosis, cellular differentiation, transformation of cells, and vascular contraction [1–4]. We have shown that angiotensin (Ang) II via activation of AT_1_ receptors increases the expression of MAP kinases in primary cultures of rat astrocytes [5–7]. ERK1/2 MAP kinases were shown to mediate Ang II-induced astrocyte growth and Ang II-induced c-Fos and c-Myc expression [5, 6, 8]. We have also established that Ang II induces the phosphorylation of stress-activated protein kinase/Jun-terminal kinase (SAPK/JNK) MAP kinases leading to cellular proliferation in cultured rat astrocytes, an effect that was also mediated by the AT_1_ Ang receptors [7]. Our findings suggest that Ang II signals through these two different MAP kinase pathways in astrocytes.

More recently, we showed that Ang III also induces the phosphorylation of ERK1/2 and SAPK/JNK MAP kinases in these cells [9, 10]. Moreover, Ang III was equipotent to Ang II in causing these MAP kinases phosphorylation and occurred via interaction with the Ang AT_1_ receptor. Ang III also induced astrocyte growth, however, not to a similar extent as Ang II [9].

Similar to our intracellular findings, *in vivo* studies have established that both peptides have similar physiologically relevant effects. For example, intracerebroventricular (ICV) injection of Ang II or Ang III caused a similar dose-dependent increase in blood pressure. Since Ang II is quickly cleaved by aminopeptidase A (APA) into Ang III, the true effector was unknown [11, 12]. In spontaneously hypertensive rats (SHR), injection of both peptides caused a prolonged blood pressure response compared to controls. However, pretreatment with bestatin, an aminopeptidase B (APB) inhibitor, potentiated and prolonged the elevated blood pressure response to Ang III in SHR [13]. Since bestatin inhibits the breakdown of Ang III, these findings suggest that Ang III was a key player in the blood pressure response elicited by the activation of the renin angiotensin system. 

Findings from several studies have implicated Ang III as the active peptide in the central nervous system. Reaux et al. [14] showed that preventing Ang II conversion to Ang III using EC33 (a selective APA inhibitor) blocked the pressor response of exogenous Ang II in rats, suggesting that the conversion of Ang II to Ang III is required to increase blood pressure in these animals. Moreover, ICV injection of EC33 caused a dose-dependent decrease in blood pressure, while ICV injection of PC18 (an APB inhibitor) increased blood pressure, an effect that was prevented by pretreatment with the AT_1_ receptor antagonist, losartan [14]. These findings and ours suggest that Ang III is a major effector peptide of the brain renin angiotensin system, and this peptide is involved in central control of blood pressure. In addition, these findings and others implicate Ang III, not Ang II, as the active peptide of the central renin angiotensin system leading to the putative “Ang III hypothesis.” Therefore, in this study, we used specific APA inhibitors to establish whether Ang II-mediated phosphorylation of ERK1/2 and SAPK/JNK MAP kinases in astrocytes was a result of Ang II conversion to Ang III. This is highly possible since APA is expressed in astrocytes [15] and neurons [16, 17]. As shown by others, inhibition of this enzyme has attenuated certain effects of Ang II. These studies were conducted in brainstem and cerebellum astrocytes to allow correlation of the current results with our previous findings [9, 10]. Moreover, astrocytes are the main source of brain angiotensinogen [18], the Ang II and Ang III precursors, and thus are ideal brain-derived cells to study the signaling pathways of these peptides. 

## 2. Materials and Methods

### 2.1. Materials

Tissue culture supplies such as Dulbecco's Modified Eagles Medium (DMEM)/F12 (1 : 1), fetal bovine serum (FBS), antibiotic solution, and trypsin/EDTA were purchased from VWR (Grand Island, NY, USA). Ang II and Ang III were obtained from Bachem (Torrance, CA, USA). The APA inhibitor glutamate phosphonate (4-amino-4-phosphonobutyric acid, GluP) was generously supplied by Dr. Robert Speth (Nova Southeastern University, FL, USA), while amastatin (AMA) was purchased from Calbiochem (La Jolla, CA, USA). ^3^H-Thymidine (2000 Ci/mmole) was purchased from MP Biomedicals (Solon, OH, USA). The phosphospecific ERK1/2 antibody, the phosphospecific SAPK/JNK antibody (Tyr751), the ERK1/2 antibody, and the SAPK/JNK antibody were purchased from Cell Signaling Technology (Beverly, MA, USA). Protein measurement supplies, gel electrophoresis, and Western blotting supplies including BCA protein reagents, acrylamide, ECL chemiluminescent reagents, and nitrocellulose membrane were purchased from either GE Health Care (Piscataway, NJ, USA) or Biorad Laboratories (Hercules, CA, USA) or from Pierce Biotechnology (Rockford, IL, USA). All other chemicals were purchased from either VWR international (Suwannee, GA, USA) or Sigma (St. Louis, MO, USA). 

### 2.2. Preparation of Astrocytes

Timed, pregnant Sprague-Dawley rats were obtained from Charles River Laboratories (Wilmington, MA, USA) and maintained in the ALAAC-accredited animal facility of Nova Southeastern University. Primary cultures of astrocytes were prepared from the brainstem and cerebellum of 2-3-day-old neonatal pups by physical dissociation as previously described [6, 8, 19]. Cells were maintained in DMEM/F12 with 10% FBS, 100 *μ*g/mL penicillin, and 100 units/mL streptomycin at 37°C in a humidified CO_2_ incubator (5% CO_2_ and 95% air). Cultures were fed every 3 to 4 days until confluent. Confluent monolayers were placed in DMEM/F12 containing 10 mM HEPES, pH 7.5, 10% FBS, and antibiotics and shaken overnight to remove oligodendrocytes. Astrocytes were detached with trypsin/EDTA (0.05% trypsin, 0.53 mM EDTA), replated at a ratio of 1 to 10, and grown to about 85% confluence prior to use. Isolated cells showed a positive immunoreactivity with an antibody against glial fibrillary acidic protein and negative immunoreactivity with markers for neurons, fibroblasts, or oligodendrocytes. 

### 2.3. Cell Treatments

Cultured brainstem and cerebellum astrocytes growing on 100 mm culture dishes were made quiescent by a 48-hour treatment with serum-free media. Astrocytes were pretreated with either 100 *μ*M GluP or 10 *μ*M AMA for 15 minutes. Following the pretreatments, the cells were subsequently stimulated with 100 nM Ang II or 100 nM Ang III for 10 minutes. For comparative purposes, cells were also incubated with the inhibitors alone. Basal and stimulated levels of the MAP kinases were determined in the presence of DMSO in experiments involving AMA. The concentrations of the GluP and AMA were not cytotoxic based on measurements of the mitochondrial uptake of the tetrazolium dye, 3-(4,5-dimethylthiazol-2-yl)-2,5-diphenyltetrazolium bromide (MTT), to its insoluble formazan form (data not shown). Cell lysates from all experiments were prepared by washing the astrocyte monolayers with phosphate-buffered saline containing 0.01 mM NaVO_4_ to prevent the dephosphorylation of activated phosphorylated proteins. Cells were solubilized in supplemented lysis buffer (100 mM NaCl, 50 mM NaF, 5 mM EDTA, 1% Triton X-100, 50 mM Tris-HCl, 0.01 mM NaVO_4_, 0.1 mM PMSF, 0.6 *μ*M leupeptin, and pH 7.4) for 30 minutes on ice. The supernatants were clarified by centrifugation (12000 ×g for 10 minutes, 4°C), and the protein concentrations were subsequently measured using the BCA method. 

### 2.4. Western Blot Analysis

Western blotting protocols to determine ERK1/2 phosphorylation and JNK phosphorylation were previously described [7, 10]. Essentially, solubilized proteins were separated in 10% polyacrylamide gels and transferred to nitrocellulose membranes. Nonspecific binding to the membranes was prevented by treating the membranes with 5% Blotto and probing with either the specific activated phosphorylated form of the ERK1/2 antibody or the activated phosphorylated form of the SAPK/JNK antibody. After incubating with the primary antibodies, the membranes were probed with goat anti-rabbit antibody coupled to horseradish peroxidase, the immunoreactive bands visualized using ECL reagents, and the data quantified by densitometry. To quantify protein loading, membranes were also probed with antibodies for the ERK1/2 protein and SAPK/JNK protein. The immunoreactive bands were visualized using ECL reagents and quantified by densitometry.

### 2.5. Measurement of DNA Synthesis

Subconfluent monolayers of cells growing in 24-well culture dishes were made quiescent by 48-hours treatment with serum-free media. Individual wells were then pretreated for 15 minutes with 100 *μ*M GluP or 10 *μ*M AMA. Subsequently, the cells were treated with either 100 nM Ang II or 100 nM Ang III for 48 hours. For comparative purposes, some wells did not receive any peptides or were treated with DMSO (the vehicle for AMA) or were only treated with the inhibitors alone. ^3^H-Thymidine (0.25 Ci/mL culture medium) was added during the last 24 hours of treatment. Newly synthesized DNA was precipitated with 5% TCA, dissolved in 0.25 N NaOH, and quantified by liquid scintillation spectrometry as previously described [20]. 

### 2.6. Statistical Analysis

All data are expressed as the mean ± SEM of 6 or more experiments, as indicated. *t*-tests or repeated measures of one-way analysis of variance (ANOVA) with Dunnett's posttest were used to compare treatment groups with groups treated with no chemicals or those treated with the inhibitors, using PRISM (GraphPad). The criterion for statistical significance was set at *P* < 0.05. 

## 3. Results

### 3.1. Effect of APA Inhibitors on ERK1/2 MAP Kinase Phosphorylation by the Peptides

**Figure 1:**
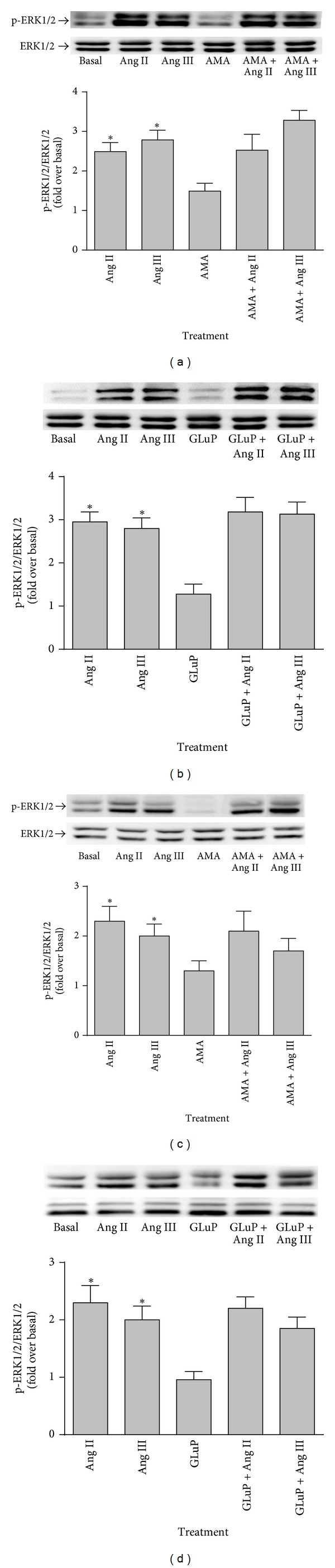
Effect of AMA and GluP on Ang II- and Ang III-induced ERK1/2 protein phosphorylation. Quiescent monolayers of brainstem ((a) and (b)) or cerebellum ((c) and (d)) astrocytes were pretreated with 10 *μ*M AMA or 100 *μ*M GluP for 15 minutes. The cells were subsequently stimulated with 100 nM Ang II or 100 nM Ang III for 10 minutes. Phosphorylated-ERK1/2 immunoreactive protein levels were measured by Western blot analysis using an antibody specific for the phosphorylated form of ERK1/2. Protein loading was quantified using the ERK1/2 protein antibody. The data were analyzed by densitometry, and the amount of phosphorylation was calculated as the fold increase over basal in the presence of vehicle. Each value represents the mean ± SEM of preparations of brainstem and cerebellum astrocytes from 6 or more litters of neonatal rat pups. *denotes that *P* < 0.05 as compared to basal levels for ERK1/2 expression in astrocytes prepared from the brainstem and the cerebellum.

In previous studies, we showed that Ang II and Ang III interact with Ang AT_1_ receptors to significantly increase ERK1/2 MAP kinase phosphorylation to a similar extent [10]. To determine whether the effects observed with Ang II were due to its conversion to Ang III, cerebellar and brainstem astrocytes were pretreated for 15 minutes with 10 *μ*M AMA or 100 *μ*M GluP. The cells were subsequently stimulated with either 100 nM Ang II or 100 nM Ang III. As shown in Figure 1, both Ang II and Ang III significantly and equipotently induced ERK1/2 MAP kinase phosphorylation in brainstem astrocytes. The APA inhibitors AMA (Figure 1(a)) and GluP (Figure 1(b)) were ineffective in preventing Ang II- and Ang III-induced ERK1/2 phosphorylation. Similarly, in cerebellar astrocytes, both peptides induced ERK1/2 MAP kinase phosphorylation, effects that were not affected by pretreatment with the APA inhibitors (Figures 1(c) and 1(d)). These findings suggest that the two peptides directly induced phosphorylation of this MAP kinase pathway.

### 3.2. Effect of APA Inhibitors on SAPK/JNK MAP Kinase Phosphorylation by the Peptides

**Figure 2:**
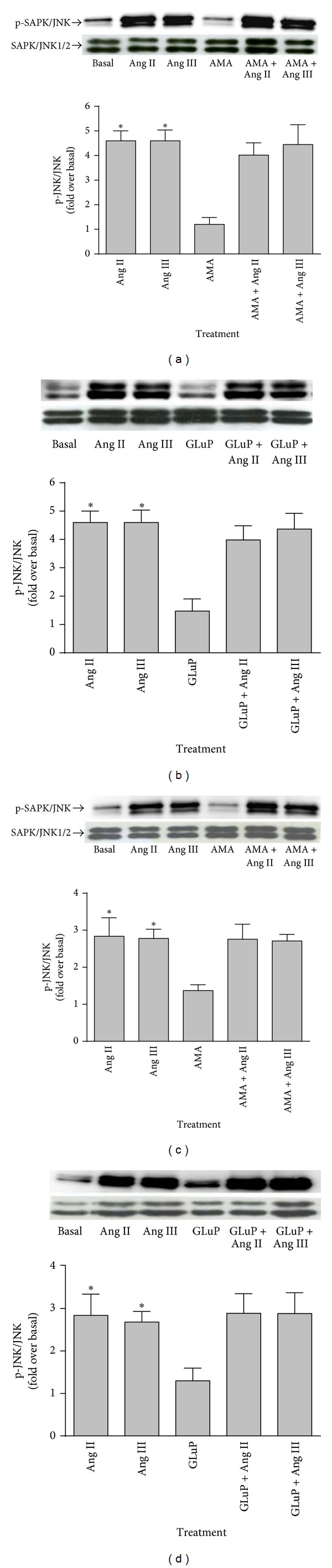
Effect of AMA and GluP on Ang II- and Ang III-induced SAPK/JNK protein phosphorylation. Quiescent monolayers of brainstem ((a) and (b)) and cerebellum ((c) and (d)) astrocytes were pretreated with 10 *μ*M AMA or 100 *μ*M GluP for 15 minutes. The cells were subsequently stimulated with 100 nM Ang II or 100 nM Ang III for 10 minutes. Phosphorylated-SAPK/JNK immunoreactive protein levels were measured by Western blot analysis using an antibody specific for the phosphorylated form of SAPK/JNK. Protein loading was quantified using the SAPK/JNK protein antibody. The data were analyzed by densitometry, and the amount of phosphorylation was calculated as the fold increase over basal in the presence of vehicle. Each value represents the mean ± SEM of preparations of brainstem and cerebellum astrocytes from 6 or more litters of neonatal rat pups. *denotes that *P* < 0.05 as compared to basal levels for SAPK/JNK expression in astrocytes prepared from the brainstem and the cerebellum.

As shown previously [9] and in Figure 2, Ang II and Ang III via interactions with the Ang AT_1_ receptor induced SAPK/JNK MAP kinase phosphorylation similarly in brainstem astrocytes. Pretreatment with the APA inhibitors AMA (Figure 2(a)) and GluP (Figure 2(b)) failed to prevent Ang II-mediated and Ang III-mediated SAPK/JNK MAP kinase phosphorylation. In cerebellar astrocytes, the two peptides were equipotent in inducing SAPK/JNK MAP kinase phosphorylation (Figures 2(c) and 2(d)). Both APA inhibitors were unable to prevent Ang II-induced and Ang III-induced SAPK/JNK MAP kinase phosphorylation in cerebellar astrocytes as well (Figures 2(c) and 2(d)). Interestingly, brainstem astrocytes were more sensitive to the effects of both peptides, exhibiting higher levels of SAPK/JNK phosphorylation as compared to cerebellum astrocytes. In addition, our findings also suggest that both peptides have direct effects to induce SAPK/JNK MAP kinase in brainstem and cerebellum astrocytes. 

### 3.3. Effects of APA Inhibitors on Ang Peptide Astrocyte DNA Synthesis

**Figure 3:**
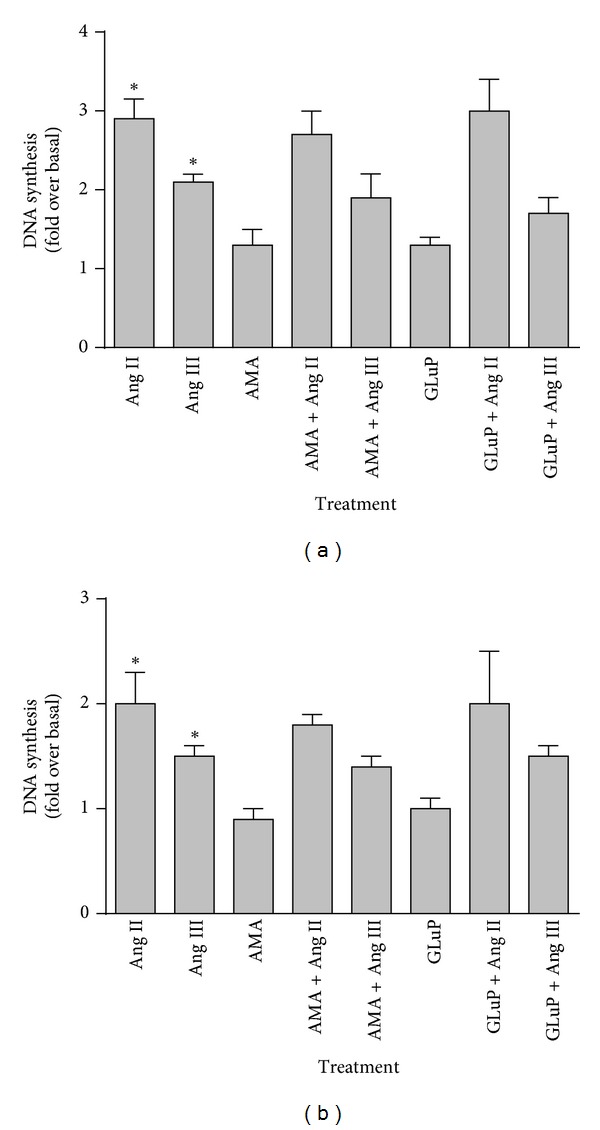
Effect of AMA and GluP on Ang II- and Ang III-induced DNA synthesis. Quiescent monolayers of brainstem (a) and cerebellum (b) astrocytes were incubated for 48 hours with 100 nM Ang II or Ang III in the presence and absence of 10 *μ*M AMA or 100 *μ*M GluP. During the last 24 hours of treatment, ^3^H-Thymidine was added and DNA synthesis measured as described. Cells were also treated with the inhibitors alone. Each value represents the mean ± SEM of preparations of brainstem and cerebellum astrocytes from 6 or more litters of neonatal rat pups. *denotes that *P* < 0.05 as compared to basal levels of DNA synthesis.

Both Ang II and Ang III have been shown to induce cellular proliferation of astrocytes [10]. To determine whether inhibition of APA affected the mitotic effects of Ang II and Ang III, brainstem and cerebellar astrocytes were pretreated with the APA inhibitors (10 *μ*M AMA and 100 *μ*M GluP) followed by stimulation with the peptides. As shown in Figure 3, both Ang II and Ang III induced cellular proliferation of brainstem and cerebellum astrocytes. However, Ang II was more potent at inducing astrocyte proliferation in both brainstem and cerebellum astrocytes, a finding that we observed previously [9, 10]. The APA inhibitors AMA and GluP (Figures 3(a) and 3(b)) were ineffective at preventing the proliferative effects of Ang II and Ang III, suggesting that both peptides had direct effects on cell proliferation. 

## 4. Discussion

We and others have established that the angiotensin peptides Ang II and Ang III have important cellular effects in the central nervous system. However, in the intact brain, controversy still surrounds the synthesis, degradation, and physiological effects of Ang II, the primary peptide produced by the renin angiotensin system. One such controversy is known as the “Ang III Hypothesis.” The premise of this theory is that the physiologically relevant peptide in the brain that binds to and activates the AT_1_ receptor is Ang III, not Ang II. This putative “Ang III Hypothesis” suggests that Ang II is converted to Ang III in order to activate Ang AT_1_ receptors [14, 21–25]. Studies have shown that centrally produced Ang II is rapidly degraded by several enzymes (primarily APA), leading to the accumulation of Ang III [26]. It is the degradation of Ang II to Ang III by APA that is the most documented breakdown pathway for Ang II and is the focus of most studies. Since the metabolism of Ang II is relatively rapid, this has helped to fuel the Ang III Hypothesis. 

There are a few studies that support the putative Ang III hypothesis. Most of these studies have focused on using APA inhibitors such as AMA to block Ang II conversion to Ang III, then measuring effects of the initial Ang II treatment on blood pressures of normotensive and hypertensive animals [11–14]. A few studies have also focused on the Ang receptor(s) that Ang III interacts with to cause effects on blood pressure. An *in vitro* study by Harding et al. [22] showed that Ang III was more potent than Ang II at eliciting responses from paraventricular nucleus receptors isolated from normal rats and SHR. The Ang II response was blocked by the addition of AMA, the selective APA inhibitor, and was ineffective at preventing the Ang III response [22].

There are a number of studies that refute the theory that Ang II conversion to Ang III is necessary for AT_1_ receptor binding. Kokje et al. [27] showed that aminopeptidase-resistant Ang II analogs, when ICV-adminiistered, caused pressor and dipsogenic activities similar to or greater than Ang II. In addition, there were no differences in Ang II, its aminopeptidase-resistant analogs, and Ang III initial peak pressor responses, findings that do not support a requisite formation of Ang III to elicit a response. It has also been established that ^125^I-Ang II can bind to brain AT_1_ receptors, an effect that does not require conversion to ^125^I-Ang III [28]. 

In the current studies, we sought to determine whether some of the effects that we have previously observed with Ang II are modulated in the presence of the APA inhibitors, AMA and GluP. AMA was selected since it is an APA inhibitor that is used by many investigators to study Ang II metabolism [26]. GluP was selected since it has recently received much attention as a direct APA inhibitor with a better selectivity for APA [28]. This enzyme was selected as a target for inhibition since it is the major enzyme involved in Ang II conversion to Ang III [26, 29]. 

In these studies, we showed that Ang III and Ang II had similar effects to induce ERK1/2 and SAPK/JNK MAP kinases phosphorylation, effects that we have previously observed [9, 10]. Blocking the ability of APA to convert Ang II to Ang III did not significantly affect the ability of Ang II to induce phosphorylation of these MAP kinases. In addition, both Ang II and Ang III induced astrocyte proliferation although Ang II was more potent, an effect we have observed previously. Further, blocking the activity of APA failed to prevent the proliferative effects of both peptides. Overall, our findings suggest that Ang II has direct effects to stimulate MAP kinases and to induce astrocyte growth since preventing its conversion to Ang III did not affect the ability of the peptide to induce MAP kinase phosphorylation or its ability to cause astrocyte proliferation. These findings fail to support the Ang III hypothesis and suggest that in astrocytes, Ang II has agonistic and mitogenic properties of its own. Most importantly, these studies also support a role for Ang III as a central peptide. 

Ang III agonistic properties were observed a number of years ago [30]; however, the relevance of this peptide as a key player in the renin angiotensin system is not yet appreciated. In astrocytes, our studies support mitogenic properties of Ang III since it directly activates ERK1/2 and SAPK/JNK MAP kinases and causes astrocyte growth [9, 10]. Most importantly, Ang III-induced astrocyte proliferation occurred via the MAP kinase pathways suggesting an important role for these pathways in Ang III effects [9, 10]. It is notable that in most instances, Ang III agonistic effects in the brain are similar to Ang II. Both peptides have been shown to induce similar central-mediated increases in blood pressure or mean arterial pressure [30, 31] and equivalent central effects on thirst and sodium appetite [32]. Centrally administered Ang III was as potent as Ang II in causing pressor and renal effects in rats on normal and high sodium diets [33]. Ang II and Ang III induced the central expression of the transcription factors c-Fos, c-Jun, and Krox-24 with the same efficacy [34]. Peripherally, Ang III was equipotent to Ang II with respect to blood pressure increases, aldosterone secretion, and renal functions [35]. On the other hand, we have shown that the ability of Ang III to cause proliferation of astrocytes was differentiated from Ang II, exhibiting a similar effect as Ang II at lower doses. At the higher doses of the peptides, astrocytes were more responsive to Ang II [10]. Thus, our studies and others have shown that Ang II and Ang III may have equivalent effects on several physiological processes but differential effects on others. Overall, our findings suggest a relevant physiological role of both Ang II and Ang III in the body, in particular in the brain. 

It has also been suggested that the APA inhibitors may be acting as agonist/antagonist of the AT_1_ receptor [26]. Thus, in the presence of the inhibitors, the AT_1_ receptor responses would be modulated. However, in our hands, pretreating with the APA inhibitors had no effect on both Ang II and Ang III to induce the MAP kinases phosphorylation, and they did not affect the proliferative effects of both peptides. Since both Ang II and Ang III responses are mediated by interaction with the AT_1_ receptor, these inhibitors are apparently not acting in an agonist/antagonist capacity in our system. The specificity and the efficacy of the APA inhibitors used in this study may be questionable and/or controversial. However, our findings are credible since most of the studies that are used to support the Ang III Hypothesis use an APA inhibitor (mostly AMA) to prevent Ang II conversion to Ang III [14, 22], suggesting that the results of our current study are directly comparable to others. 

Even though a central renin angiotensin system is widely accepted, there are still few controversies that surround its existence, a major one being the Ang III Hypothesis. Our findings suggest that in brainstem and cerebellum astrocytes, both Ang II and Ang III are direct potent central mitogens and that their activities are unaffected by APA inhibitors. Thus, *in vitro*, these findings suggest that both peptides are important modulators of the actions of the brain renin angiotensni system. The relevance of these findings must be corroborated *in vivo* since cell cultures cannot give an overall picture of all actions of these peptides in the brain. It is important that we establish the major peptides that govern the actions of the central renin angiotensin system due to the significance of this system in many physiological and disease processes. Thus, our findings are essential in establishing the relevance of both Ang II and Ang III as direct acting endogenous central peptides.
